# Irinotecan and its metabolite SN38 inhibits procollagen I production of dermal fibroblasts from Systemic Sclerosis patients

**DOI:** 10.1038/s41598-021-97538-3

**Published:** 2021-09-09

**Authors:** J. Lapoirie, L. Tran, L. Piazza, C. Contin-Bordes, M. E. Truchetet, F. Bonnet

**Affiliations:** 1 Service de Médecine Interne et Maladies Infectieuses, Hôpital Saint-André, CHU de Bordeaux, 1 rue Jean Burguet, 33000 Bordeaux, France; 2 Aquitaine Science Transfert, 33400 Talence, France; 3 Direction de La Recherche Clinique et de L’Innovation, CHU de Bordeaux, 33000 Bordeaux, France; 4 Département d’Immunologie et d’Immunogénétique, and CNRS-UMR5164 ImmunoConcEpT, Université de Bordeaux, 33000 Bordeaux, France; 5 Service de Rhumatologie, Hôpital Pellegrin, CHU de Bordeaux, 33000 Bordeaux, France; 6 Université de Bordeaux, INSERM U1219, Bordeaux Population Health, 33000 Bordeaux, France

**Keywords:** Cell biology, Immunology, Medical research, Rheumatology

## Abstract

Systemic sclerosis (SSc) is a rare autoimmune connective tissue disease characterized by a microangiopathy and fibrosis of the skin and internal organs. No treatment has been proved to be efficient in case of early or advanced SSc to prevent or reduce fibrosis. There are strong arguments for a key role of topo-I in the pathogenesis of diffuse SSc. Irinotecan, a semisynthetic derivative of Camptothecin, specifically target topo-I. This study was undertaken to evaluate the effects of noncytotoxic doses of irinotecan or its active metabolite SN38 on collagen production in SSc fibroblasts. Dermal fibroblasts from 4 patients with SSc and 2 healthy donors were cultured in the presence or absence of irinotecan or SN38. Procollagen I release was determined by ELISA and expression of a panel of genes involved in fibrosis was evaluated by qRT-PCR. Subcytotoxic doses of irinotecan and SN38 caused a significant and dose-dependent decrease of the procollagen I production in dermal fibroblasts from SSc patients, respectively − 48 ± 3%, *p* < 0.0001 and − 37 ± 6.2%, *p* = 0.0097. Both irinotecan and SN38 led to a global downregulation of genes involved in fibrosis such as COL1A1, COL1A2, MMP1 and ACTA2 in dermal fibroblasts from SSc patients (respectively − 27; − 20.5; − 30.2 and − 30% for irinotecan and − 61; − 55; − 50 and − 54% for SN38). SN38 increased significantly CCL2 mRNA level (+ 163%). The inhibitory effect of irinotecan and its active metabolite SN38 on collagen production by SSc fibroblasts, which occurs through regulating the levels of expression of genes mRNA, suggests that topoisomerase I inhibitors may be effective in limiting fibrosis in such patients.

## Introduction

Systemic sclerosis (SSc) is a multisystem connective tissue disorder featured by vascular injury, autoimmune background and fibrosis affecting the skin and internal organs. The pathophysiology of SSc remains incompletely understood but involves a complex network of interactions between the microvascular system, activation of autoimmune processes, and chronic activation of fibroblasts leading to the deregulated production of extracellular matrix (ECM) proteins, predominantly type I collagen, and finally fibrosis ^1,2^.

There is no specific therapeutic for SSc. The use of immunosuppressive agents is frequent in case of extensive skin or visceral involvement, in particular cyclophosphamide, mycophenolate mofetil and methotrexate. In the last few years, many treatments have been evaluated in SSc : targeted immunotherapies such as belimumab ^3^, pomalidomide ^4^ or tocilizumab ^5^; anti-fibrotic therapies such as pirfenidone ^6^ and nintedanib ^7^ which are approved for use in patients with idiopathic pulmonary fibrosis ^8^ and riociguat ^9^ which is approved in pulmonary arterial hypertension ^10^ for its vascular benefit. Neither of these treatments has been shown to have a benefit on overall and respiratory mortality, or on skin and lung fibrosis compared to placebo, except a small but significant effect on full vital capacity of nintedanib.

An international consortium implemented a therapeutic trial evaluating autologous hematopoietic stem cell transplantation (HSCT) versus intravenous pulse cyclophosphamide in diffuse cutaneous SSc ^11^. Patients in the HSCT group experienced better long-term overall survival but higher mortality in the first year than those treated with cyclophosphamide alone.

Thus, treatment for fibrosis in SSc is still an unmet medical need.

Anti-topoisomerase I (Topo-I) antibodies are frequently associated to diffuse cutaneous SSc and to severe systemic and visceral involvement (pulmonary fibrosis, cardiac, digestive and joint involvement, scleroderma renal crisis) ^12^. Human DNA topo-I is a 765-amino-acid nuclear enzyme involved in topological changes of DNA structure. It plays key roles in alleviating the topological stresses that arise during DNA replication and transcription by nicking, relaxing, and re-ligating the double-stranded DNA structure. There are strong arguments for a key role of topo-I in the pathogenesis of scleroderma since topo-I is overexpressed in SSc ^13^. Indeed, selective oxidation of DNA topo-I induces SSc in the mouse and is associated with anti-topo-I antibodies occurrence, endothelial production of reactive oxygen species and fibroblast hyperproliferation, showing that the specific oxidation of topo-I directly participates in the pathogenesis of SSc ^14^. Moreover, topo-I is able to bind specifically to the cell surface of fibroblasts ^15^ and then induce anti–topo-I autoantibodies binding, trigger the adhesion and activation of monocytes ^16^ and activate T-lymphocytes ^17^. These cells could further lead to the initiation and maintenance of an inflammatory cascade, stimulating the fibrosis that is characteristic of SSc.

Irinotecan, a semisynthetic derivative of the natural alkaloid Camptothecin, specifically target topo-I. The conversion of the water-soluble irinotecan prodrug into its active metabolite SN38 allows DNA cleavage but inhibits subsequent ligation by trapping the enzyme on DNA, generating cytotoxic protein-linked DNA breaks ^18,19^. Irinotecan is currently a major anticancer drug, contributing to the treatment of patients with advanced colon cancers and other solid tumors including non-small cell lung cancer, pancreatic and biliary tract cancers, advanced gastric and cervical cancer ^20–23^.

We observed surprisingly that irinotecan or an irinotecan active metabolite administered in a patient suffering from both rectum adenocarcinoma and SSc with anti topo-I antibodies to high level (141 IU/mL, normal range 0–29) can improve fibrosis, and in particular skin involvement (personal data).

Regarding our case report and the analysis of the literature, we hypothesize that irinotecan or its active metabolite SN38 may have an effect on the inhibition of the production of collagen by SSc fibroblasts and could represent a specific treatment for diffuse SSc. The aim of this study was to evaluate the effect of subcytotoxic doses of irinotecan and SN38 on collagen production by dermal fibroblasts obtained from patients with diffuse SSc.

## Methods

### Study population

The subjects were patients with diffuse cutaneous SSc and healthy volunteers. Patients with diffuse SSc were included in the context of the VISS (Vasculopathy and Inflammation in Systemic Sclerosis) biomedical research project funded in 2012 and approved by the institutional ethical committee of Bordeaux University Hospital (CPP, 2012-A00081-42). Informed consent was obtained from all participants and research have been performed in accordance with the Declaration of Helsinki. All participants satisfied the classification criteria proposed by the American College of Rheumatology (ACR) and the European League Against Rheumatism (EULAR) 2013 ^24^ and suffered from diffuse SSc with positive anti topo-I antibodies. Punch biopsy specimen of affected mid-forearm skin were obtained. The control group were healthy donors (HD) that underwent plastic surgery (brachioplasty). None of the healthy individuals had dermatological disorders or were under immunosuppressive agents/glucocorticoids.

### Cell culture

Fibroblasts were obtained from skin lesion biopsy samples from 2 HD and 4 SSc patients. Skin biopsies were digested with 0.1% type Ia collagenase at 37 °C for 2-h. Adherent cells were grown in DMEM medium containing 2 mM of L-glutamine, 50 U/mL penicillin, 50 µg/ml streptomycin and 10% foetal bovine serum (FBS) (cDMEM). To keep homogeneity between fibroblasts they were used between the third and sixth passages ^25^.

### Determination of procollagen I release

Dermal fibroblasts were monolayer plated at 20,000 cell per well in DMEM 10% FBS supplemented with 20 µg/mL of vitamin C in 96-well plates. 24 h before the experiment, medium was switched to DMEM 0% FBS supplemented with 20 µg/mL of vitamin C (L-Ascorbic acid). As demonstrated in the literature, IC_50_ inhibition of proliferation values for irinotecan were between 2 and 3.8 µM ^26^, the first concentration producing significant cytotoxicity being 32 µM ^27^, whereas IC_50_ values for SN38 were 0.1 µM ^28^. In consequence, cells were treated for 48 h with irinotecan (0.006, 0.6, 6 or 40 µg/mL, corresponding to 0.01, 0.1, 1 or 64 µM) or SN38 (0.01, 0.06, 0.1 or 0.3 µg/mL, corresponding to 0.025, 0.15, 0.25 or 0.76 µM) in DMEM 1% FBS supplemented with 20 µg/mL of vitamin C. Supernatants were collected to evaluate the procollagen I release level by ELISA kit (ref MK101, supplier Takara) according to the manufacturer’s instructions. Each reaction was performed in triplicate. The level of procollagen I release was presented as fold change compared with the procollagen I release by the same non-treated fibroblasts arbitrary fixed at 100%.

### Quantitative real-time PCR

Dermal fibroblasts were plated in DMEM 10% FCS in 24-well plates. 24 h before the experiment, medium was switched to DMEM 0% FBS. Cells were treated for 24 h with irinotecan (2 or 20 µg/mL, corresponding to 3.2 µM or 32 µM) or SN38 (0.04 or 0.4 µg/mL, corresponding to 0.1 or 1 µM) in DMEM 1% FBS. Supernatants were removed and plates were freezed at − 80°c. Total fibroblasts RNA from each well-plate were extracted with NucleoSpin® RNA Plus kit (Macherey–Nagel) according to the manufacturer’s instructions. The quantity and quality of RNA were evaluated by capillary electrophoresis (Bioanalyzer 2100, Agilent). RNA was used for cDNA synthesis using the Transcriptor Reverse Transcriptase kit (Roche). qPCR reactions were run on a Light Cycler (Roche Molecular Systems Inc.) according to the manufacturer’s instructions. The specific primer pairs for each gene are described in the supplementary material table 1. Each reaction was performed in triplicate. The relative gene expression levels of COL1A1, COL1A2, MMP1, TIMP1, CCL2 and ACTA2 were calculated using the ΔΔCt method. Target mRNA levels were normalized to stable housekeeping gene GAPDH (Glyceraldehyde-3-phsophate dehydrogenase). Expression of the genes in the different treated conditions was presented as fold change compared with the gene expression of the same non-treated fibroblasts arbitrary fixed at 100%.

### Statistical analysis

Statistical analyses were performed using GraphPad Prism (La Jolla, CA). Statistical significance was evaluated using paired t-test. Data values are expressed as means ± SEM. A p-value < 0.05 was considered statistically significant.

### Ethics approval and consent to participate

The VISS (Vasculopathy and Inflammation in Systemic Sclerosis) biomedical research project has been approved by the institutional ethical committee (CPP, 2012-A00081-42). All participants provided written informed consent before inclusion.

## Results

### Irinotecan decreases procollagen I production of dermal fibroblasts in a dose-dependent manner

**Figure 1:**
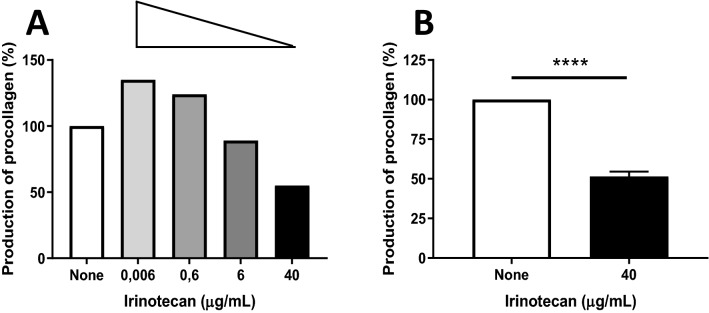
Analysis of irinotecan effect on procollagen I release of dermal SSc fibroblasts. The level of procollagen I release was evaluated after 48 h in dermal fibroblasts from SSc patients incubated with irinotecan. A comparison of procollagen I release level was done between non-treated condition and treated conditions. **(A)** Data from a representative experiment showing a dose-dependent effect on the procollagen I production of SSc fibroblasts after incubation with 4 different doses of irinotecan (i.e. 0.006 µg/ml, 0.6 µg/ml, 6 µg/ml, 40 µg/ml). **(B)** Cumulative data from 3 independent experiments showing a decrease of procollagen I production in SSc fibroblasts incubated with irinotecan (40 µg/ml). ****p < 0.0001 using paired t-test.

The main manifestation of systemic sclerosis is the overproduction of ECM and especially type I collagen. Therefore, we assessed whether irinotecan directly impacts the procollagen I production of dermal fibroblasts. Four concentrations of irinotecan (0.006 µg/ml, 0.6 µg/ml, 6 µg/ml, 40 µg/ml) were assessed and compared to a non-treated condition. All the doses used were under a cytotoxic threshold determined on fibroblasts by preliminary toxic analysis at concentration of irinotecan of 200 µg/ml (supplementary data tables 2a). Irinotecan treatment during 48 h resulted in a dose-dependent decrease of procollagen I production levels in SSc and HD fibroblasts. In a representative experiment, the highest dose of irinotecan (40 µg/mL) resulted in about 45% inhibition of procollagen I production levels in SSc fibroblasts (Fig. 1A). The effect of irinotecan at the concentration of 40 µg/mL on the release of procollagen I protein was then assessed in 4 SSc patients and 2 HD dermal fibroblasts. Three independent experiments were done. Incubation with 40 µg/mL irinotecan during 48 h significantly decreased the release of procollagen I protein in SSc patients (− 48.5 ± 3 for dermal fibroblasts of SSc patients treated with irinotecan vs. the non-treated condition, Fig. 1B * p* <  0.0001, paired t-test). No difference was noted between SSc patients and HD dermal fibroblasts (data not shown).

### SN38 decreases procollagen I production of dermal fibroblasts in a dose-dependent manner

**Figure 2:**
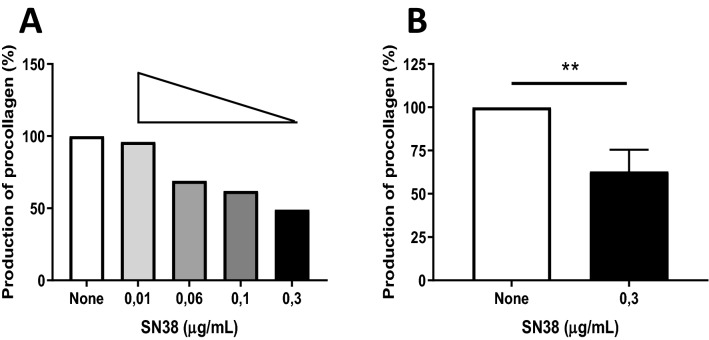
Analysis of SN38 effect on procollagen I release of dermal SSc fibroblasts. The level of procollagen I release was evaluated after 48 h in dermal fibroblasts from SSc patients incubated with SN38. A comparison of procollagen I release level was done between non-treated condition and treated conditions. **(A)** Data from a representative experiment showing a dose-dependent effect on the procollagen I production of SSc fibroblasts after incubation with 4 different doses of SN38 (i.e. 0.01 µg/ml, 0.06 µg/ml, 0.1 µg/ml, 0.3 µg/ml). **(B)** Cumulative data from 3 independent experiments showing a decrease of procollagen I production in SSc fibroblasts incubated with SN38 (0.3 µg/ml). ** *p* < 0.01 using paired t-test.

We assessed whether an irinotecan active metabolite, SN38, directly impacts the procollagen I production of dermal fibroblasts. Four concentrations of SN38 (0.01, 0.06, 0.1 or 0.3 µg/mL), were assessed and compared to a non-treated condition. All the doses used were under a cytotoxic threshold determined on fibroblasts by preliminary toxic analysis at concentration of SN38 of 1 µg/ml (2.55 µM) (supplementary data tables 2b). SN38 treatment during 48 h resulted in a dose-dependent decrease of procollagen I production levels in SSc and HD fibroblasts. In a representative experiment, the highest dose of SN38 (0.3 µg/mL) resulted in about 50% inhibition of procollagen I production levels in SSc fibroblasts (Fig. 2A). The effect of SN38 at the concentration of 0.3 µg/mL on the release of procollagen I protein was then assessed in 4 SSc patients and 2 HD dermal fibroblasts. Three independent experiments were done. Incubation with 0.3 µg/mL SN38 during 48 h significantly decreased the release of procollagen I protein in SSc patients (− 37 ± 6.2 for dermal fibroblasts of SSc patients treated with SN38 vs. the non-treated condition, Fig. 2B * p* = 0.0097, paired t-test). No difference was noted between SSc patients and HD dermal fibroblasts (data not shown).

### Subcytotoxic doses of irinotecan decrease mRNA expression of a panel of genes involved in fibrosis

**Figure 3:**
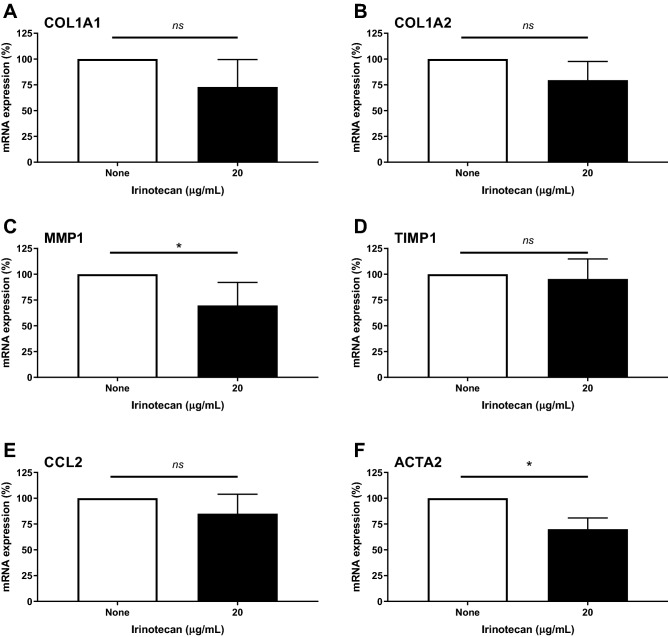
Analysis of irinotecan effect on mRNA expression of genes involved in fibrosis in dermal SSc fibroblasts. Relative mRNA expression of a panel of genes involved in fibrosis (from A to F: COL1A1, COL1A2, MMP1, TIMP1, CCL2 and ACTA2) in dermal fibroblasts obtained from 4 systemic sclerosis patients treated with irinotecan (20 µg/mL), in comparison to a non-treated condition. The non-treated condition was arbitrary fixed at 100%. Analysis of qRT-PCR reactions normalized to stable housekeeping gene GAPDH (Glyceraldehyde-3-phosphate dehydrogenase). A level above 100% corresponds to a gene overexpression in the treated condition and a level below 100% corresponds to a gene downregulation in the treated condition. Mean ± SEM, ns: non significant, * p < 0.05 using paired t-test.

To assess the effect of irinotecan on mRNA levels of a panel of gene related to fibrosis, 4 SSc patients and 2 HD dermal fibroblasts were incubated with or without irinotecan during 24 h. As reported before, the two concentrations (2 µg/mL and 20 µg/mL) of irinotecan used were not cytotoxic on the fibroblasts and led to a global downregulation of genes involved in fibrosis in SSc patients, except for the metallopeptidase inhibitor TIMP1 gene (supplementary data Fig. 1). Incubation with the highest evaluated dose of irinotecan (20 µg/mL) during 24 h significantly decreased mRNA expression of MMP1 (− 30.25 ± 11, *p* = 0.05, paired t-test) and ACTA2 (− 30 ± 5.4, *p* = 0.0117, paired t-test) in dermal fibroblasts of SSc patients treated with irinotecan vs. the non-treated condition. Although incubation with 20 µg/mL irinotecan during 24 h decreased mRNA expression of COL1A1 (− 27 ± 13.25), COL1A2 (− 20.5 ± 9) and CCL2 (− 15 ± 9.4) in dermal fibroblasts of SSc patients treated with irinotecan *vs*. the non-treated condition, these differences were not statistically significant (Fig. 3). Finally, irinotecan had no effect on mRNA expression of TIMP1 (− 4.5 ± 9.6). No difference was noted between SSc patients and HD dermal fibroblasts (data not shown).

### Subcytotoxic doses of SN38 decrease mRNA expression of a panel of genes involved in fibrosis

**Figure 4:**
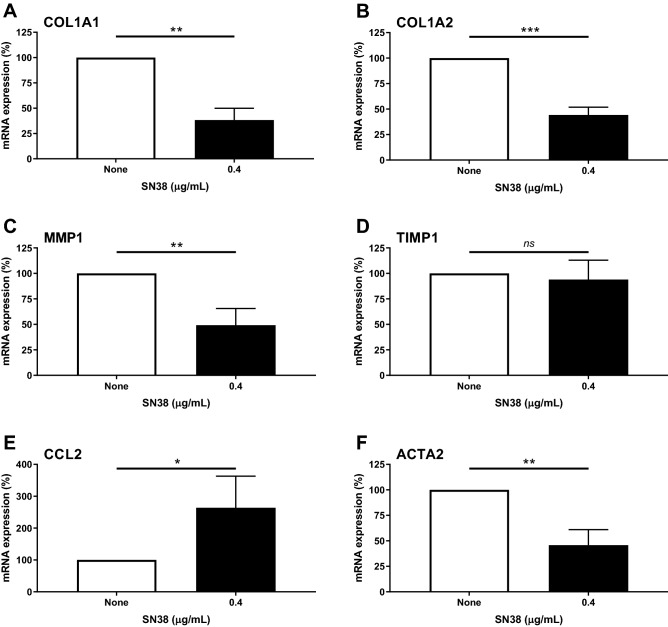
Analysis of SN38 effect on mRNA expression of genes involved in fibrosis in dermal SSc fibroblasts. Relative mRNA expression of a panel of genes involved in fibrosis (from A to F: COL1A1, COL1A2, MMP1, TIMP1, CCL2 and ACTA2) in dermal fibroblasts obtained from 4 systemic sclerosis patients treated with SN38 (0.4 µg/mL), in comparison to a non-treated condition. The non-treated condition was arbitrary fixed at 100%. Analysis of qRT-PCR reactions normalized to stable housekeeping gene GAPDH (Glyceraldehyde-3-phsophate dehydrogenase). A level above 100% corresponds to a gene overexpression in the treated condition and a level below 100% corresponds to a gene downregulation in the treated condition. Mean ± SEM, ns: non significant, *p < 0.05, ** p < 0.01, *** p < 0.001 using paired t-test.

To assess the effect of SN38, an active irinotecan metabolite, on mRNA levels of a panel of fibrosis gene, 4 SSc patients and 2 HD dermal fibroblasts were incubated with or without SN38 during 24 h. As reported before, the two concentrations (0.04 µg/mL and 0.4 µg/mL) of SN38 used were not cytotoxic on the fibroblasts and led to a global downregulation of genes involved in fibrosis in SSc patients (supplementary data Fig. 1), except for TIMP1 and CCL2 genes (supplementary data Fig. 2). Incubation with the highest evaluated dose of SN38 (0.4 µg/mL) during 24 h significantly decreased mRNA expression of COL1A1 (− 61.75 ± 5.8, *p* = 0.0018, paired t-test), COL1A2 (− 55.75 ± 3.7, *p* = 0.0007, paired t-test), MMP1 (− 50.75 ± 8,1, *p* = 0.0084, paired t-test) and ACTA2 (− 54.25 ± 7.6, *p* = 0.0057, paired t-test) in dermal fibroblasts of SSc patients treated with SN38 vs. the non-treated condition (Fig. 4). SN38 had no effect on mRNA expression of TIMP1 (− 6 ± 9.4). In contrast, incubation with 0.4 µg/mL SN38 during 24 h significantly increased mRNA expression of CCL2 (+ 163.8 ± 49.6, *p* = 0.04, paired t-test) in dermal fibroblasts of SSc patients treated with SN38 vs. the non-treated condition. No difference was noted between SSc patients and HD dermal fibroblasts (data not shown).

These results suggest that irinotecan and its active metabolite SN38 decrease in part the levels of expression of some genes mRNA involved in SSc fibrosis.

## Discussion

In this study, we showed that both irinotecan, a clinically approved derivative of camptothecin, and its active metabolite SN38 significantly inhibit the synthesis of collagen by dermal fibroblasts from patients with diffuse SSc, regulating the expression of genes involved in fibrosis.

Fibrosis associated to SSc occurs when inappropriate tissue remodeling leads to excessive ECM deposition, predominantly type I collagen by fibroblasts, resulting from abnormal interactions between endothelial cells, lymphocytes/monocytes and fibroblasts. In our study we did not observed an increased production of procollagen 1 in SSc fibroblasts when compared to controls (data not shown). To our knowledge, only 2 studies ^29,30^ previously showed a potent selective inhibitory effect of camptothecin on collagen synthesis in dermal fibroblasts from SSc patients.

The effect of irinotecan and SN38 was assessed on the procollagen I production of dermal fibroblasts of SSc patients and HD. A dose-dependent effect was observed, and we thus tested the highest doses (40 µg/ml for irinotecan and 0.3 µg/ml for SN38). Our results demonstrated that irinotecan and SN38 significantly inhibits the synthesis of procollagen I by dermal fibroblasts from patients with SSc. According to Czuwara-Ladykowska et al. ^30^, no difference was noted between dermal fibroblasts from SSc patients and HD suggesting, like nintedanib ^31^, that Irinotecan/SN38 may interact with procollagen production through the same pathway in SSc and non-SSC fibroblasts.

The effect of irinotecan and SN38 was then assessed on a panel of 6 key genes involved in ECM remodeling: COL1A1 and COL1A2 which are ECM components, MMP1 which is one of the major matrix metalloproteinase involved in degradation of the ECM, TIMP1 which is a natural inhibitor of most of the known matrix metalloproteinases, CCL2 which is a pro-fibrotic gene coding for an inflammatory chemokine ^32^ and ACTA2 (alpha-SMA) which is a pro-fibrotic gene expressed specifically in myofibroblasts. Both incubation with irinotecan and SN38 decreased the expression of COL1A1, COL1A2, MMP1 and ACTA2 genes in dermal fibroblasts of HD and SSc patients, with a statistically significant effect for SN38. No effect was observed on TIMP1 gene expression. Surprisingly, CCL2 gene expression was significantly increased after incubation with SN38. However, the profibrotic role of the CCL2/CCR2 axis is controversial. Indeed, CCL2 had no direct effects on collagen production by fibroblasts in various studies ^33,34^. Interestingly, Kalderen et al. ^35^ demonstrated that CCL2 induces anti-fibrotic signaling in fibroblasts independently of CCR2, suggesting that CCL2 may have diametrically opposite effects on fibroblasts depending on the in vivo setting in health and disease.

In the field of immunology, in addition to anti-fibrotic effects, immunomodulation of peripheral blood mononuclear cells (PBMC) activity could complete the efficacy of irinotecan. In SSc, camptothecin showed immunosuppressive activity, down-regulating the expression of IL-2 receptor and then reducing the proliferation of PBMC and natural killer cells activity ^29^. Recent publication regarding the interest of irinotecan in murin models of Systemic Lupus Erythematosus have suggested that the topo I inhibitor irinotecan is able to suppress lupus nephritis in NZB/NZW mice ^36^, and to suppress both lupus nephritis and lupus-like skin lesions in MRL/lpr mice ^37^. Spleen cell populations demonstrated a decrease of approximately one third of the total number of B cells as well as lower B and T cell activity in the irinotecan-treated groups. Further experiments showed that extremely low concentrations (50 times lower than the dose applied for chemotherapy in humans) of irinotecan still suppressed lupus nephritis and prolonged survival in NZB/NZW mice ^38^. However, B cell activation was lower only in the group treated with the highest dose of irinotecan.

In the context of advanced SSc, the safety profile of irinotecan seems acceptable. Indeed, SSc has the highest disease-related mortality of all autoimmune connective tissue diseases with a standard mortality ratio of 3.5, a median survival time after diagnosis of 11 years, and an absolute survival at 5 years of 77.9%. Cardiopulmonary involvement (lung fibrosis, pulmonary arterial hypertension and heart involvement) accounts for the majority of the increased mortality in SSc ^12^. In the recent therapeutic trial evaluating autologous HSCT versus intravenous pulse cyclophosphamide in diffuse cutaneous SSc ^11^, a total of 156 patients were randomly assigned to receive HSCT (Hematopoietic stem cell transplantation) (n = 79) or cyclophosphamide (n = 77). During a median follow-up of 5.8 years, 53 events occurred: 22 in the HSCT group (19 deaths and 3 irreversible organ failures) and 31 in the control group (23 deaths and 8 irreversible organ failures). This underlines the high level of mortality and of morbidity of diffuse scleroderma, whatever the immunosuppressive treatment in a selected population.

Irinotecan is a well-known drug used for more than 12 years in patients with cancer, including patients with metastatic cancer in palliative care. In the field of oncology, SN38 is described as 100 to 1000-fold more potent as a topo-I inhibitor than irinotecan ^39,40^, which is consistent with our results. To note, the highest doses we used during our experiments (40 µg/ml irinotecan) correspond to the doses currently recommended in oncology (100 to 350 mg/m2 every week to every third week) and are known to have poor side-effects ^41–43^, even combined with other anticancer drugs such as paclitaxel ^44^. The adverse events observed in patients treated at the recommended dose were modest and manageable, mainly represented by neutropenia and diarrhea. Regarding these data, we hypothesize that the safety profile of irinotecan in SSc patients would be comparable to that of other immunosuppressive drugs used in the context of scleroderma such as cyclophosphamide, mycophenolate mofetil, or rituximab. Therefore, the benefit/risk ratio of this treatment appears favourable.

Finally, the main limitation of our study is the small number of patients included. Moreover, since irinotecan and SN38 are topo-I inhibitor, we can not exclude that they may have impact on other protein synthesis and that the effect observed may be non-specific. However, regarding the poor prevalence of SSc (7.2–33.9 per 100,000 individuals in Europe) with high mortality rate (Ten-year survival at 65–73% in Europe) ^45^ and the lack of effective treatment, we believe that our results can be of interest in the field of SSc and deserve further studies.

In conclusion, this study showed that inhibition of Topo-I activity by noncytotoxic doses of irinotecan or its active metabolite SN38 could reverse the pro-fibrotic phenotype of fibroblasts from patients with diffuse SSc. It paves the road to a potential new treatment for diffuse SSc or other fibrotic diseases with installed fibrosis, where effective treatment is still lacking. Further studies will be needed to determine the effect and the safety of irinotecan in patients with diffuse SSc.

## Supplementary Information

Supplementary Information.

## Data Availability

The datasets used and/or analysed during the current study are available from the corresponding author on reasonable request.

## Abbreviations

Topo-I: Anti-topoisomerase I
ECM: Extracellular matrix
FBS: Foetal bovine serum
HD: Healthy donors
HSCT: Hematopoietic stem cell transplantation
PBMC: Peripheral blood mononuclear cells
SSc: Systemic sclerosis
