# Supplementary material for: Irinotecan and its metabolite SN38 inhibits procollagen I production of dermal fibroblasts from Systemic Sclerosis patients
Source: Sci Rep. 2021 Sep 9;11:18011. doi: 10.1038/s41598-021-97538-3 (PMC8429710; doi:10.1038/s41598-021-97538-3)

**Supplementary data**

**Table 1**: specific primer pairs used for each gene for qPCR reactions

| **gene** | **Abbreviation** | **Gene Bank** | **primer sens** | **primer antisens** | **cDNA bp** |
| --- | --- | --- | --- | --- | --- |
| Collagen, type I, alpha 1 | COL1A1 | NM_000088 | CGATGGATTCCAGTTCGAGTA  (SEQ ID NO. 1) | GTTTACAGGAAGCAGACAGG  (SEQ ID NO. 2) | 420 |
| Collagen, type I, alpha 2 | COL1A2 | NM_000089 | AGGTGTAAGCGGTGGTGGTTATGAC  (SEQ ID NO. 3) | CCGGATACAGGTTTCGCCAGTAGAG  (SEQ ID NO. 4) | 313 |
| Matrix metallopeptidase 1 | MMP1 | NM_002421 | ACTGCTGCTGCTGCTGTTCTG  (SEQ ID NO. 5) | TGCTTCATCACCTTCAGGGTTTCAG  (SEQ ID NO. 6) | 249 |
| TIMP metallopeptidase inhibitor 1 | TIMP1 | NM_003254 | TGCAATTCCGACCTCGTCATCAGGGC  (SEQ ID NO. 7) | AGAAACTCCTCGCTGCGGTTGTGGG  (SEQ ID NO. 8) | 215 |
| Chemokine (C-C motif) ligand 2 | CCL2 | NM_002982 | TTCTCAAACTGAAGCTCGCACTCTCGCC  (SEQ ID NO. 9) | TGTGGAGTGAGTGTTCAAGTCTTCGGAGTT  (SEQ ID NO. 10) | 348 |
| Actin, alpha 2, smooth muscle, aorta | ACTA2 | NM_001613 | AATGGCTCTGGGCTCTGTAA  (SEQ ID NO. 11) | TGGTGATGATGCCATGTTCT  (SEQ ID NO. 12) | 199 |

**Tables 2: Preliminary toxicity studies:**

We assessed in preliminary studies if irinotecan and its active metabolite, SN38 impacts the cell viability. Eight concentrations of irinotecan and SN38 were assessed on dermal fibroblasts monolayer plated in DMEM during 24h. Cell viability was assessed by MTT reduction assay and morphologic examination.

**Tables 2a: Preliminary toxicity study of irinotecan on the 6 fibroblast linear cells**

Legend: + normal population; +/- reduction density; *toxicity

**Tables 2b: Preliminary toxicity study of SN38 on the 6 fibroblast linear cells**

Legend: + normal population; +/- reduction density; *toxicity

**Figure 1: Subtoxic doses of irinotecan decrease mRNA expression of a panel of genes involved in fibrosis.**


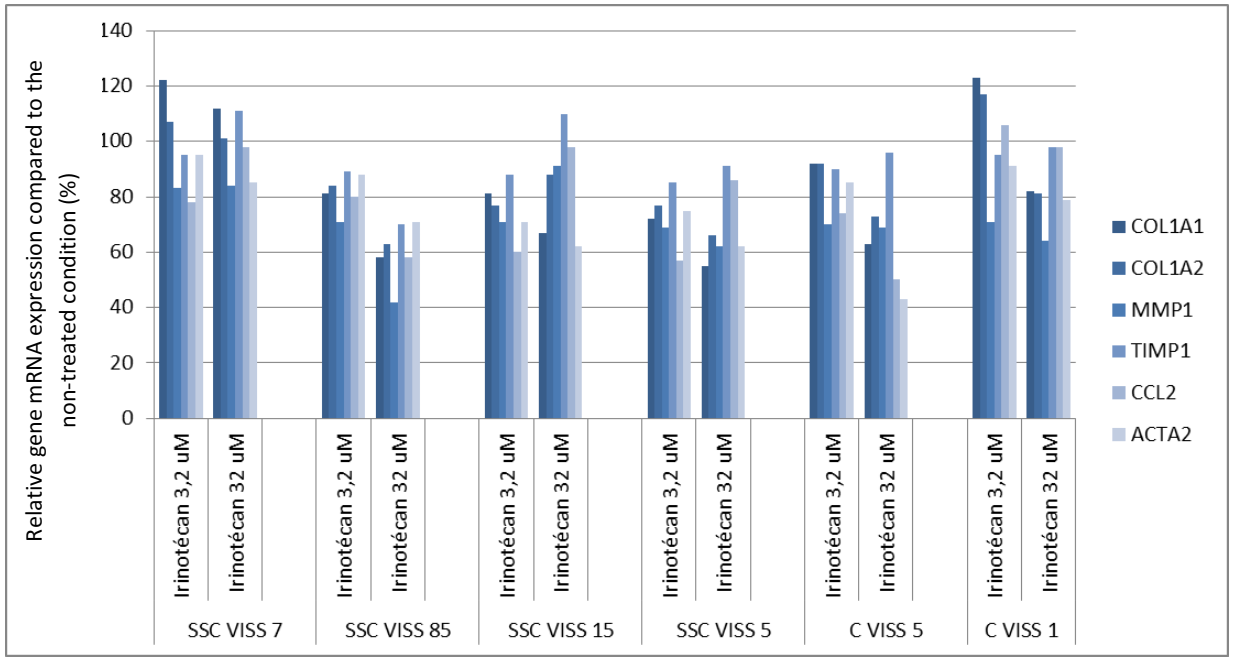


**Figure 2: Subtoxic doses of SN38 decrease mRNA expression of a panel of genes involved in fibrosis.**


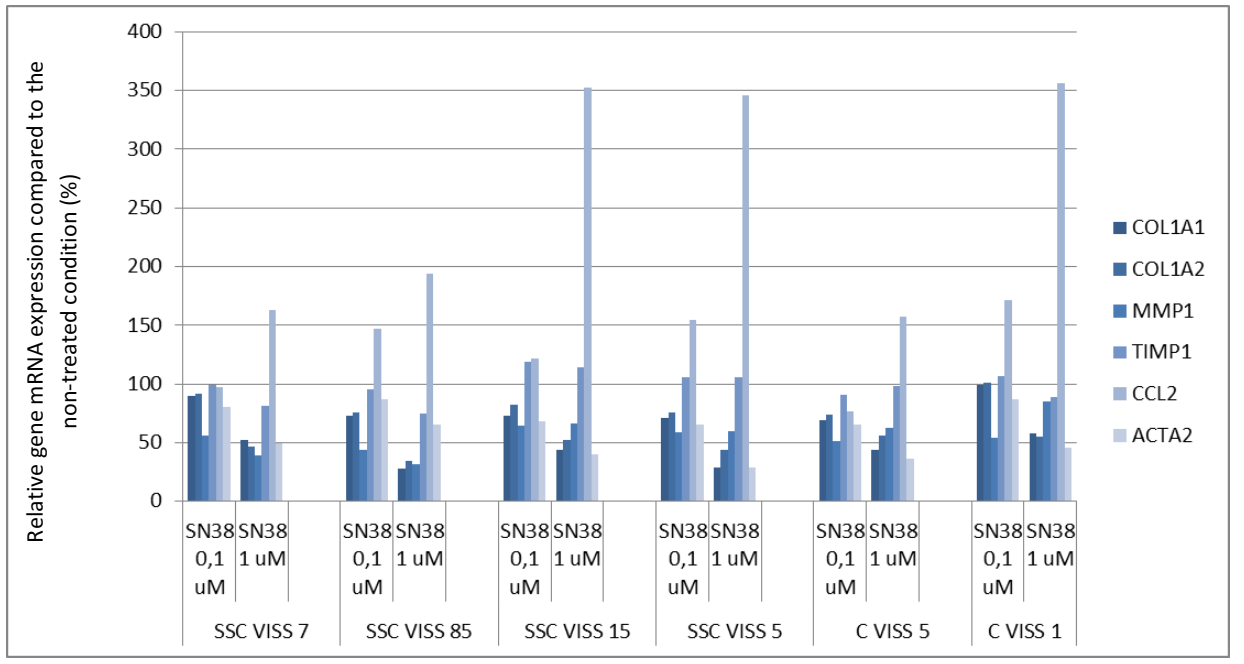

Supplement: Supplementary file 1 — Supplementary Information. [file 41598_2021_97538_MOESM1_ESM.docx]
